# Identification of differentially expressed miRNAs associated with diamide detoxification pathways in *Spodoptera frugiperda*

**DOI:** 10.1038/s41598-024-54771-w

**Published:** 2024-02-21

**Authors:** Rashmi Manohar Mahalle, Weilin Sun, Omar A. Posos-Parra, Sunghoon Jung, David Mota-Sanchez, Barry R. Pittendrigh, Keon Mook Seong

**Affiliations:** 1 Institute of Agricultural Sciences, Chungnam National University, Daejeon, Republic of Korea; 2 Department of Entomology, Center for Urban and Industrial Pest Management, Purdue University, West Lafayette, IN USA; 3 Department of Entomology, Michigan State University, East Lansing, MI USA; 4 Department of Smart Agriculture Systems, Chungnam National University, Daejeon, Republic of Korea; 5 Department of Applied Biology, College of Agriculture and Life Sciences, Chungnam National University, Daejeon, Republic of Korea

**Keywords:** Gene expression profiling, Entomology, Molecular biology

## Abstract

The fall armyworm (FAW) *Spodoptera frugiperda* is a severe economic pest of multiple crops globally. Control of this pest is often achieved using insecticides; however, over time, *S*. *frugiperda* has developed resistance to new mode of action compounds, including diamides. Previous studies have indicated diamide resistance is a complex developmental process involving multiple detoxification genes. Still, the mechanism underlying the possible involvement of microRNAs in post-transcriptional regulation of resistance has not yet been elucidated. In this study, a global screen of microRNAs (miRNAs) revealed 109 known and 63 novel miRNAs. Nine miRNAs (four known and five novel) were differentially expressed between insecticide-resistant and -susceptible strains. Gene Ontology analysis predicted putative target transcripts of the differentially expressed miRNAs encoding significant genes belonging to detoxification pathways. Additionally, miRNAs are involved in response to diamide exposure, indicating they are probably associated with the detoxification pathway. Thus, this study provides comprehensive evidence for the link between repressed miRNA expression and induced target transcripts that possibly mediate diamide resistance through post-transcriptional regulation. These findings highlight important clues for further research to unravel the roles and mechanisms of miRNAs in conferring diamide resistance.

## Introduction

The fall armyworm (FAW), *Spodoptera frugiperda* (J. E. Smith, 1797) (Lepidoptera: Noctuidae), is one of the world’s most destructive lepidopteran, infesting more than 350 plant species^1^. Geographically, it has recently spread to distant regions from its native tropical and subtropical America to Africa^2,3^ and Asia^4–8^. Over the past four decades, there has been overreliance on chemical pesticides to control FAW in agricultural fields and the pest has gradually shown varying levels of resistance to 45 active ingredients^9,10^, including the currently most widely utilized diamide insecticides^11–14^.

Diamide insecticides comprising chlorantraniliprole and flubendiamide have become increasingly popular since their commercial launch in 2006 and 2007, respectively^11,15^. These insecticides exhibit a novel mode of action, broad-spectrum insecticidal activity, and high toxicity to lepidopteran pests. However, the indiscriminate use of this class of insecticides has exacerbated the selection pressure, eventually leading to the evolution of resistance^16^. Previous studies have implicated that in addition to the ryanodine receptor gene, metabolic detoxification genes, mainly phase I reaction enzymes of P450s, phase II reaction enzymes of GSTs and UGTs, and phase III reaction enzymes of ABC transporters also contribute to the diamide resistance development^17^.

MicroRNAs (miRNAs) are a group of short non-protein-coding RNAs of about 19–24 bp in length. They are known to play a vital role in post-transcriptional gene regulation by complementary binding to coding sequences, 3′ or 5′ untranslated regions (UTRs), thereby inhibiting the mRNA translation and ultimately silencing the target gene expression^18–20^. Functional studies have described their roles in many biological processes, particularly development, metamorphosis, reproduction, immunity and insecticide resistance^20–24^.

Despite their relatively small size, miRNAs play pivotal roles as potential targets of insecticide detoxification and regulation of insecticide resistance among arthropod species. To date, several studies have identified miRNAs that are known to regulate genes that mediate insecticide resistance within insect species, especially *S*. *fr*u*giperda*. For instance, a previous study identified seven novel miRNAs using expressed sequence tags (ESTs) of *S*. *frugiperda*^25^. Another study predicted 110 differentially expressed novel miRNAs in the *S*. *frugiperda* cell line, Sf21^26^. In addition, 76 and 68 known and 139 and 171 novel miRNA candidates were noted in the two strains of *S*. *frugiperda*, corn (C) and rice (R) strains, respectively^27^. A recent research study discovered 24, 22, and 31 differentially expressed miRNAs following exposure to cyantraniliprole, spinetoram, and emamectin benzoate, respectively. The differential expression of miR-278-5p, miR-13b-3p, miR-10485-5p, and miR-10483-5p confirmed their possible involvement in insecticide resistance in *S*. *frugiperda*^28^. The role of miR-190-5p in mediating chlorantraniliprole resistance in *S*. *frugiperda* was demonstrated by regulating CYP6K2 expression^29^. Of the 30 differentially expressed miRNAs, miR‐278‐5p significantly responded following exposure to tetraniliprole (a relatively new diamide), indicating its regulatory role in *S*. *frugiperda* resistance^30^. Recent studies on the mechanisms of miRNA-regulated chlorantraniliprole resistance in *P*. *xylostella* have discovered several known and novel miRNA candidates (including miR-7a, miR-8519, miR-2b-3p and miR-14b-5p) associated with the regulation of both *PxRyR* expression and the increased enzyme detoxification^31–33^.

Furthermore, silencing target genes *CYP321A8*, *CYP321A9*, and *CYP321B1* in *S*. *frugiperda* by RNA interference revealed their critical role in insecticide detoxification by enhancing their susceptibility to chlorantraniliprole^34^. The expression profiles of miRNAs (mainly miR-282 and miR-989) targeting the cytochrome P450 expression suggested their putative functional role in imidacloprid-treated *Leptinotarsa decemlineata*^35^. According to Seong *et al*^36^, the miR-310 s cluster affects endogenous *CYP6g1* and *CYP6g2* transcript levels and is associated with DDT resistance in *Drosophila melanogaster*. Evidence shows that the differentially expressed miRNAs in response to imidacloprid (notably, smi-miR-278 and smi-miR-316) in *Sitobion miscanthi* operate as post-transcriptional regulators of *nAChRα1A* and *CYP4CJ6*, respectively^20^. miRNAs viz., novel-85, novel-191, and novel_268 were found to modulate the expression of *CYP6ER1*, carboxylesterase 1 (*CarE1*), and *NlABCG3*, respectively, which significantly altered the susceptibility of *Nilaparvata lugens* to nitenpyram^37,38^. Elevated expression of some carboxylesterase (*CarE*) genes (*PxαE14* and *PxCCE016b*) have also been reported to participate in insecticide resistance to different insecticides, including chlorantraniliprole in the field population of *Plutella xylostella*^39,40^.

GSTs are speculated to be linked with the metabolic detoxification of chlorantraniliprole through the upregulation of target transcripts in *Bombyx mori* and *P*. *xylostella*^41^. Based on their upregulated mRNA expression, some UGT genes may be associated with insect resistance to an array of insecticides (DDT, pyrethroids, carbamates, and neonicotinoids), including diamides^42^. There is evidence that ABC transporters contribute to insecticide resistance in several insects. For example, overexpression of several genes (*Mdr65*, *ABCG3*, *ABCG1*, *ABCG6*, *9* &*14*, and *NlABCG3*) was proven to be involved in resistance in *D*. *melanogaster*^43^, *B*. *tabaci*^44^, *Chilo suppressalis*^45^, *P*. *xylostella*^46^, and *N*. *lugens*^37^, respectively.

The present study generated miRNA libraries to identify differentially expressed miRNAs from insecticide-resistant and -susceptible strains of *S*. *frugiperda*. Subsequently, the inducible miRNA response to insecticide exposure in field-collected strains was investigated. This study provides insights into the potential roles of miRNAs in regulating metabolic detoxification of insecticides in *S*. *frugiperda* field populations.

## Results

### miRNA libraries and screen of the known and novel miRNAs

The sequencing data has been submitted to the Gene Expression Omnibus (GEO) at the National Center for Biotechnology Information (NCBI) under the accession number GSE233976. Using three biological replicates, we constructed six miRNA libraries from resistant and susceptible strains to identify differentially expressed miRNAs in *S. frugiperda*. The sRNA libraries yielded 290.4 million raw reads from all samples of *S. frugiperda* (Table 1). After removing low-quality reads, adaptors, and all possible contaminants, 80.18 and 67.66 million trimmed reads and 49.70 and 39.09 million unique mappable reads were retained among the triplicates of the resistant and susceptible libraries, respectively. They were further used in subsequent analyses (Table 2).

**Table 1:** Summary of read counts of small RNA sequencing from each sample.

Sample	Raw reads	Trimmed reads (%)	Mapped reads (%)	Expressed miRNAs (%)
FAW R1	62,839,424	33,555,694 (53.40)	19,850,915 (59.16)	170 (98.84)
FAW R2	57,140,734	30,458,291 (53.30)	18,925,509 (62.14)	171 (99.42)
FAW R3	49,658,398	16,164,246 (32.55)	10,929,564 (67.62)	170 (98.84)
FAW S1	33,790,521	17,703,905 (52.39)	10,302,454 (58.19)	171 (99.42)
FAW S2	46,572,391	30,067,359 (64.56)	16,548,196 (55.04)	169 (98.26)
FAW S3	40,405,220	19,897,829 (49.25)	12,246,535 (61.55)	166 (96.51)

**Table 2:** Differentially expressed miRNAs in resistant and susceptible strains of *S. frugiperda.*

miRNA Id	miRBase	miRNA sequence	log2 fold change
Spodoptera_mature_09	sfr-miR-novel-09	CCAUCCCUCACAUGAUGUAACU	1.32
Spodoptera_mature_10	sfr-miR-novel-10	GUUGAAUACCUGACAAAAUCCU	− 3.08
Spodoptera_mature_11	sfr-miR-novel-11	GUGAGUGCGUCGCCGGGCCCCG	− 1.15
Spodoptera_mature_53	sfr-miR-novel-53	UGAGUCGUUCUUAAAGUAUGCC	− 2.29
Spodoptera_mature_60	sfr-miR-novel-60	AUUCCAAUGUUCGUUAGUGAUAUA	− 1.28
Spodoptera_mature_104	sfr-miR-10460-5p	GAGCCAAUGUUCGUUAGUGAUG	− 1.56
Spodoptera_mature_134	sfr-miR-10483-2-3p	CCUGUAACUGUUCGUUCCCUU	− 1.35
Spodoptera_mature_155	sfr-miR-10465-5p	CUGGCUUGUAUUCUCGCUGCC	− 4.90
Spodoptera_mature_159	sfr-miR-10476-5p	CGAGCGAUGGUUGGAAUCCAACUGU	2.54

Algorithms in the miRDeep2 package aligned the trimmed reads to the miRNA precursors in miRBase R.22. They identified 172 conserved miRNAs from three sRNA libraries of the resistant and susceptible strains of *S. frugiperda*. Among these, 64, 42 and 3 miRNA precursors exactly matched with miRNA entries of *P*. *xylostella*, *S*. *frugiperda* and *B*. *mori*, respectively, in the miRBase R.22 database and were accepted as known miRNAs, while the remaining unmatched sequences were filtered to determine novel miRNAs using miRDeep2 software. Thus, 109 known and 63 novel miRNAs were predicted to be shared between the libraries derived from both strains (Tables S1 and S2). The length distribution of most of the sequences ranged from 19 to 25 nt, with an abundant number of typical 22 nt miRNAs, accounting for 55.23% (Fig. S2).

### Differentially expressed miRNA analysis

The comparison of normalized read count data revealed overall uniformity across triplicate libraries derived from resistant strains compared to the susceptible strains of *S. frugiperda*. However, we found nine miRNAs that showed significant differential expression between the resistant and susceptible *S. frugiperda* strains (P < 0.05 and |log2 (fold-change)|> 1) (Table 2). Specifically, seven miRNAs were significantly downregulated in the resistant strain, of which the known miRNA, sfr-miR-10465-5p, showed the highest (− 4.90 fold) downregulation, followed by sfr-miR-novel-10 (− 3.09 fold) and sfr-miR-novel-53 (− 2.29 fold). The two upregulated miRNAs in the resistant strain included one novel miRNA (sfr-miR-novel-09) exhibiting a 1.33-fold increase and one known miRNA (sfr-miR-10476-5p) with a higher expression of 2.54-fold (Table 2). The expression levels of these differentially expressed miRNAs were illustrated using volcano plots (Fig. S3). In general, the majority of the differentially expressed miRNAs were downregulated in the resistant strains compared to the susceptible strains of *S. frugiperda*. These results suggest the further necessity of validating these miRNAs to reveal their potential roles in diamide resistance.

### Diamide toxicity evaluation

Diamide toxicity in the field populations of *S. frugiperda* is summarized in Table 3. The bioassay results of chlorantraniliprole and flubendiamide showed that the LC_50_ for field populations of *S. frugiperda* were 0.26 mg/L and 1.47 mg/L, respectively. The resulting LC_50_ values of both insecticides were used to investigate the expression patterns of the differentially expressed miRNAs under diamide exposure.

**Table 3:** Toxicity analysis of *S. frugiperda* field populations in response to diamide insecticides.

Insecticide	N	LC_50_ (mg/L)	95% Fiducial limits		Slope (± SE)	χ^2^
			Lower	Upper		
Chlorantraniliprole	540	0.26	0.07	0.47	1.47 ± 0.48	0.18
Flubendiamide	630	1.47	0.24	4.27	0.80 ± 0.21	0.73

N: Number of larvae assayed; χ^2^: Chi-square value.

### Time-dependent expression profiles of miRNAs in response to diamide exposure

**Figure 1:**
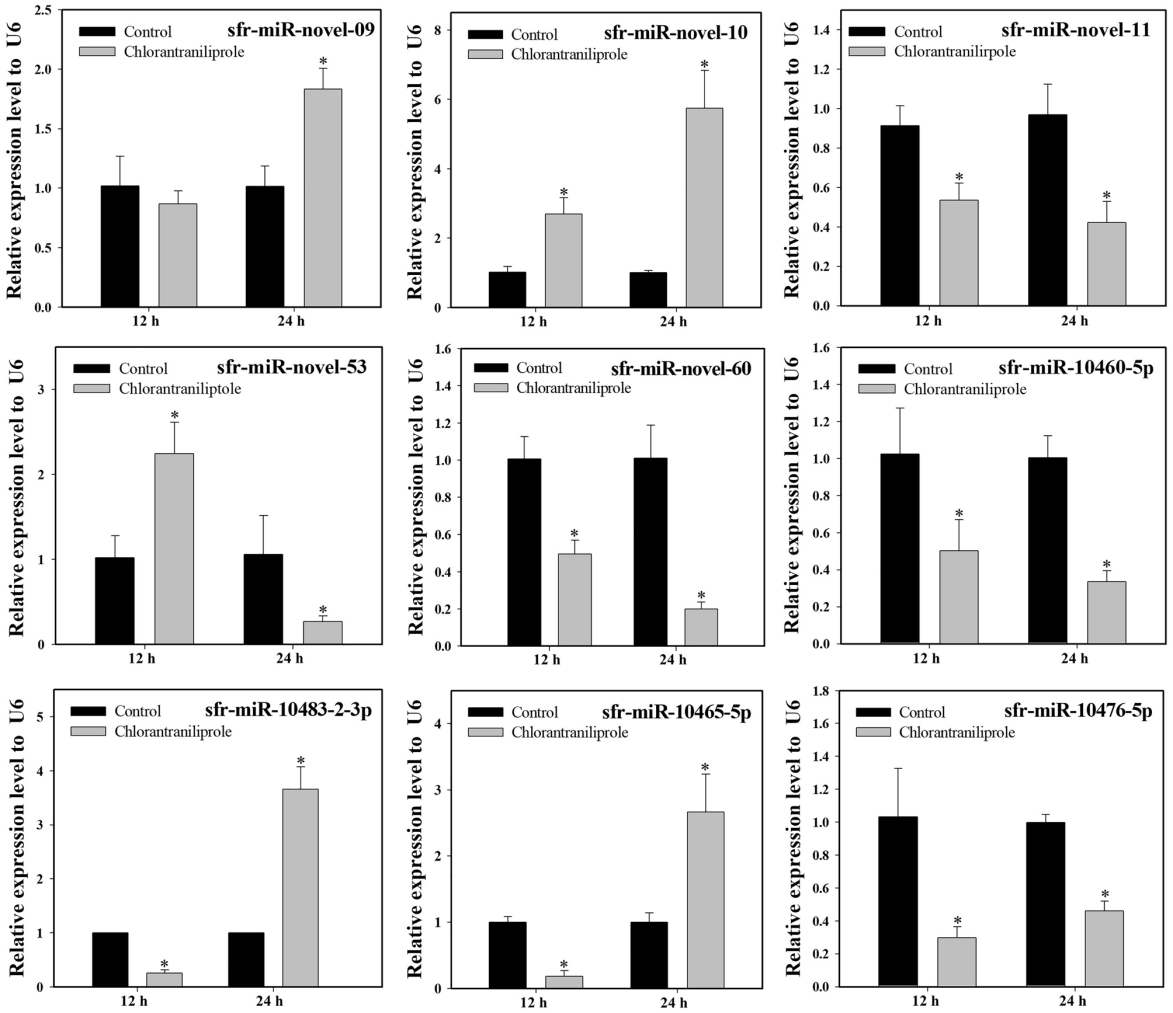
Relative expression of differentially expressed miRNAs after chlorantraniliprole treatment in the field populations of *S. frugiperda*. The expression levels were normalized by U6. Statistical significance was analysed using a paired t-test. The asterisks represent significant difference (P < 0.05).

**Figure 2:**
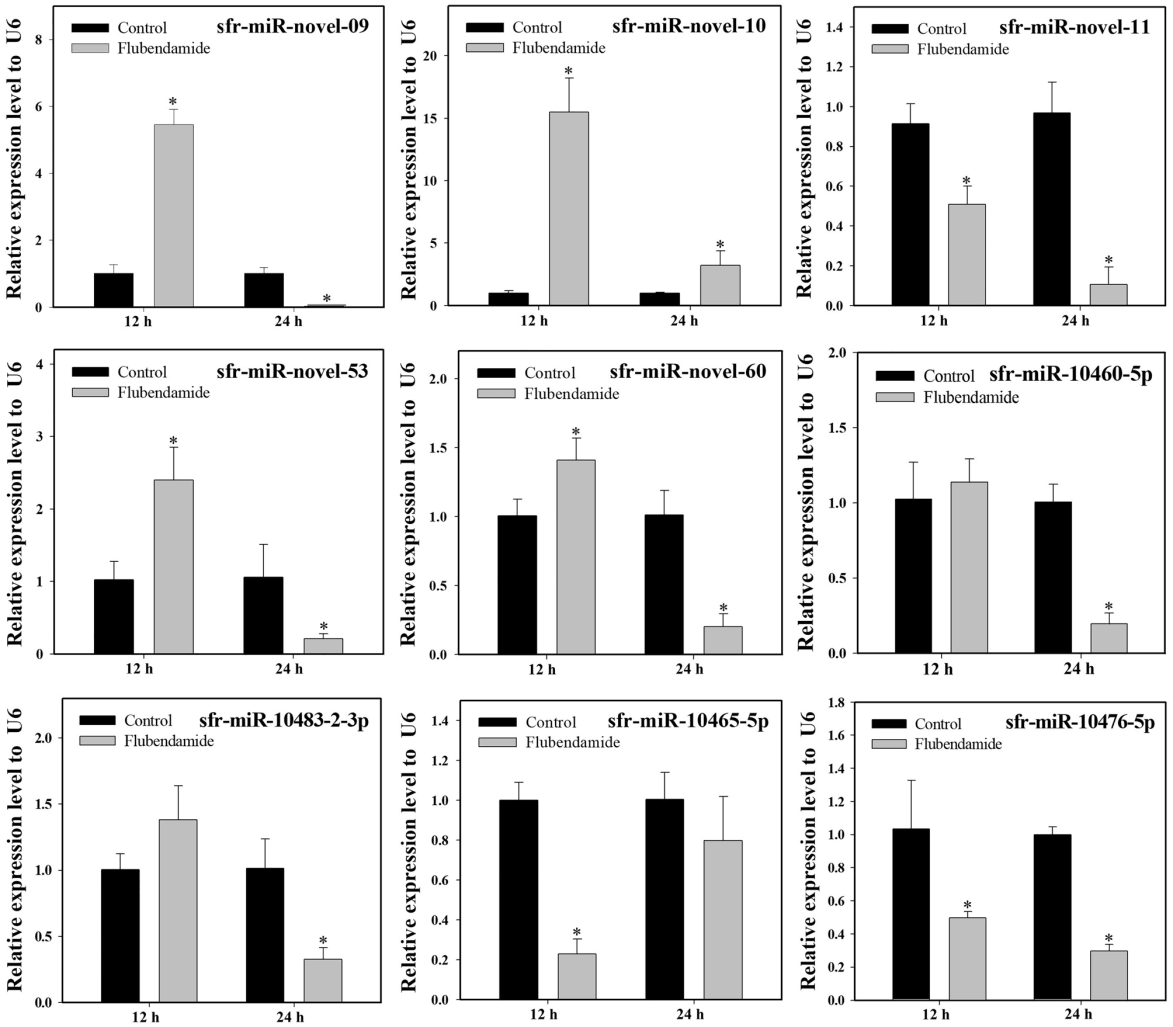
Relative expression of differentially expressed miRNAs after flubendamide treatment in the field populations of *S. frugiperda*. The expression levels were normalized by U6. Statistical significance was analysed using a paired t-test. The asterisks represent significant difference (P < 0.05).

We examined the induction or repression profiles of nine miRNAs (four known and five novel) following chlorantraniliprole and flubendiamide exposure that was shown to be differentially expressed between resistant and susceptible *S. frugiperda* strains. Most of the selected miRNAs showed similar expression patterns in response to chlorantraniliprole and flubendiamide exposure at different time points, as determined by Illumina sequencing. Compared to the acetone-treated control, three miRNAs, including sfr-miR-novel-11, sfr-miR-10460-5p and sfr-miR-10476-5p, showed consistently downregulated expression across all time points after chlorantraniliprole and flubendiamide treatments. Similarly, the expression of sfr-miR-10483-2-3p and sfr-miR-10465-5p was downregulated at 12 h, with significant upregulation at 24 h after chlorantraniliprole treatment (Figs. 1 and 2). Comparatively, sfr-miR-novel-53 showed variable expression at different time points, initially upregulated at 12 h and then downregulated after 24 h exposure to chlorantraniliprole and flubendiamide. Interestingly, sfr-miR-novel-60 was confirmed to be significantly downregulated after 12 and 24 h, and sfr-miR-novel-09 showed significant upregulation at 24 h of chlorantraniliprole treatment. However, both of these miRNAs depicted a significant upward-downward trend after flubendiamide exposure for 12–24 h. In contrast, one putative novel miRNA (sfr-miR-novel-10) was consistently upregulated at all time points following exposure to chlorantraniliprole and flubendiamide. Additionally, a few miRNAs showed no significant changes in expression (induction or repression), such as sfr-miR-novel-09 after 12 h of chlorantraniliprole treatment, sfr-miR-10460-5p, and sfr-miR-10483–2-3p after 12 h and sfr-miR-10465-5p after 24 h flubendiamide exposure (Figs. 1 and 2).

### Gene Ontology (GO) enrichment and KEGG pathway analysis of target genes

**Figure 3:**
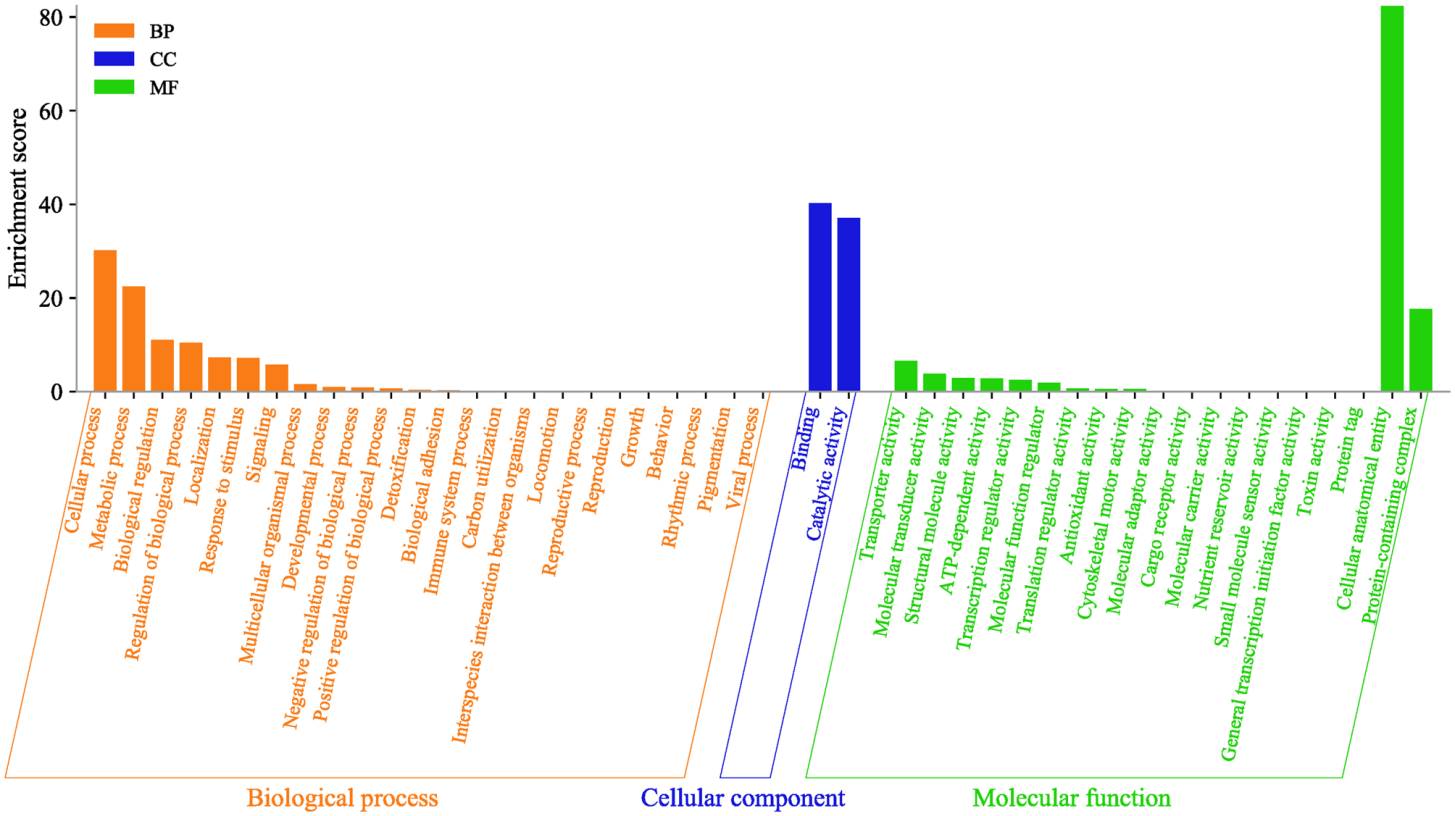
Gene ontology (GO) analysis of the most targeted genes of the differentially expressed miRNAs in *S. frugiperda.*

GO enrichment analysis was performed to understand the functional relevance of the putative target transcripts of the differentially expressed miRNAs. The GO annotations assigned a total of 55,263 target transcripts according to their involvement in biological processes (49.36%), molecular functions (30.22%), and cellular components (20.42%), comprising 45 GO terms (Fig. 3, Table S3). The highly enriched putative functions among the target genes were mainly focused on the cellular, anatomical entity and protein-containing complex in the molecular function category, followed by binding and catalytic activity represented in the cellular component category. Cellular processes, metabolic processes, biological regulation, localization, response to stimulus, and signalling were the most abundant functions in the biological process category (Table S3).

### Prediction of target genes involved in the detoxification pathway

**Figure 4:**
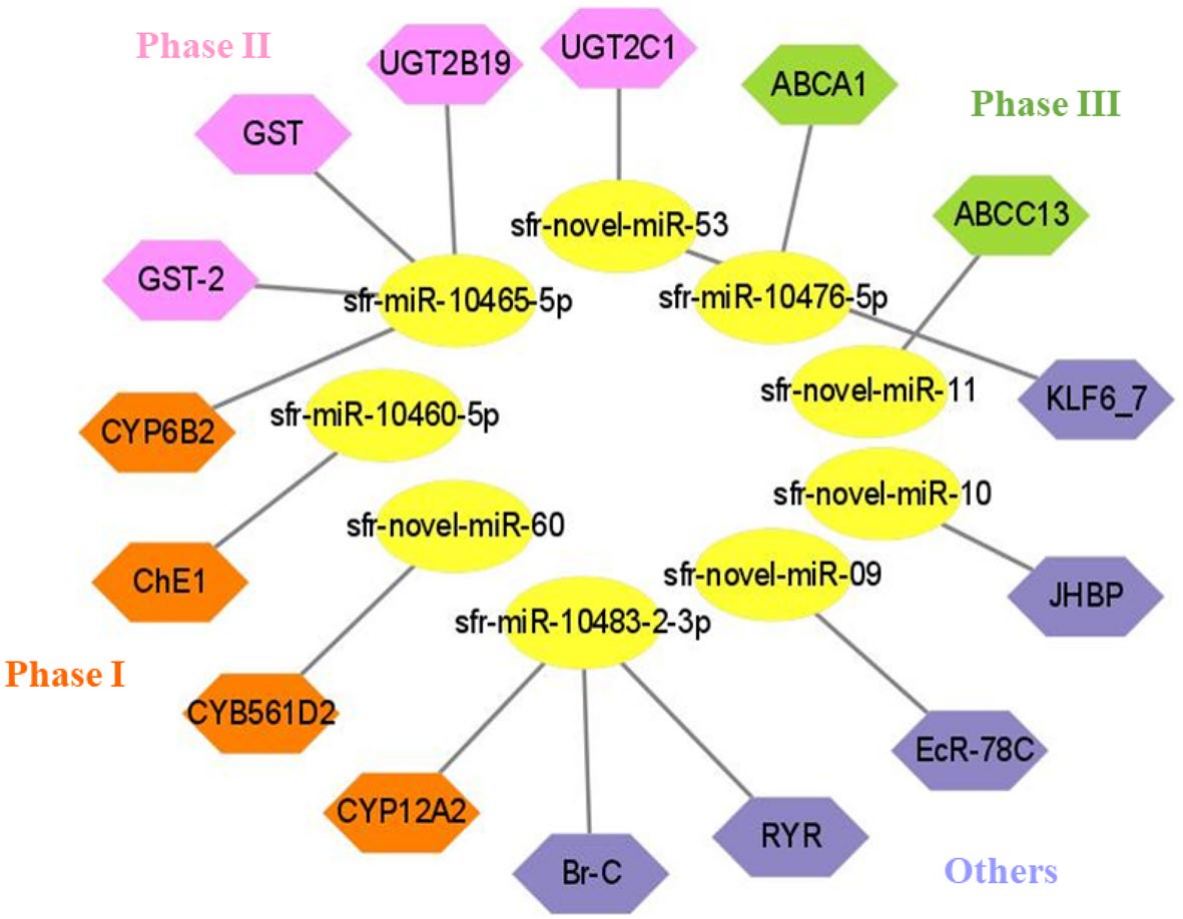
Putative target genes of differentially expressed miRNAs.

Considering only nine differentially expressed miRNAs between resistant and susceptible strains of *S. frugiperda*, potential target transcripts were predicted using the algorithms applied by the psRNATarget server (https://www.zhaolab.org/psRNATarget/). We further explored the interaction between nine differentially expressed miRNAs and their target transcripts, specifically, those genes likely to be involved in the detoxification of insecticides (Fig. 4; Table S4). Target genes encoded three-phase enzymes, including three cytochrome P450s (*CYB561D2*, *CYP6B2* and *CYP12A2*) and one cholinesterase (*ChE1*) from phase I, two UDP-glucuronosyl transferases (*UGT2B19* and *UGT2C1*) and two glutathione S-transferases (*GST* and *GST-2*) from phase II, and two ABC transporters (*ABCC13* and *ABCA1*) from phase III detoxification pathways. We also identified the ryanodine receptor (*RyR*), broad-complex core protein (*BR-C*), ecdysone-induced protein 78C-like (*EcR-78C*), Krüppel-like factor (*KLF 6_7*), and juvenile hormone binding protein-like (*JHBP*) in our transcriptome analyses (Fig. 4).

### Correlation between the expression levels of the miRNAs and their target genes

**Figure 5:**
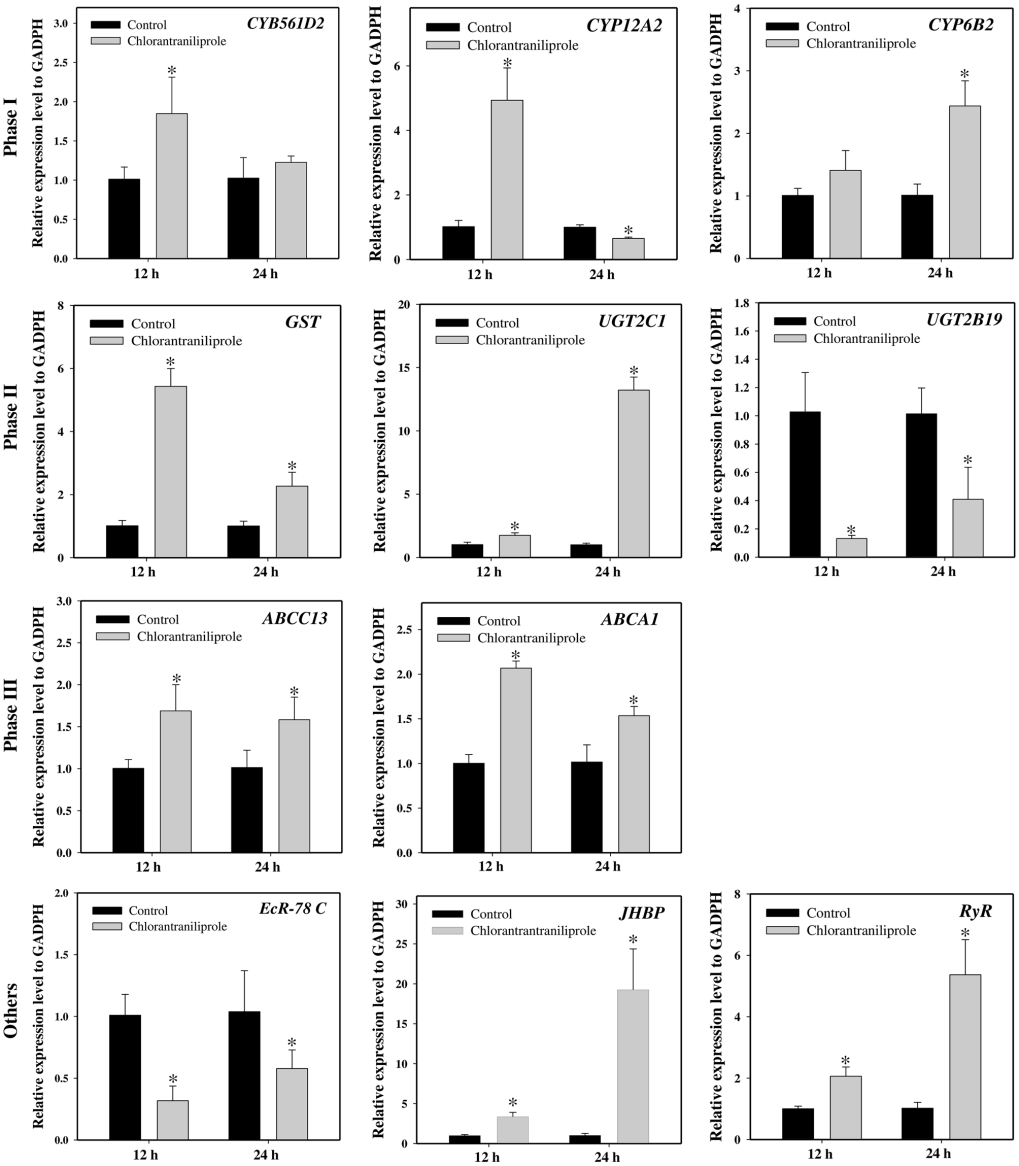
Relative expression of potential target genes of differentially expressed miRNAs after chlorantraniliprole treatment in the field populations of *S. frugiperda*. The expression levels were normalized by GADPH. Statistical significance was analysed using a paired t-test. The asterisks represent significant difference (P < 0.05).

To gain insight into the functional relationship between miRNAs and their corresponding putative targets, we estimated the expression profiles of target genes using RT-qPCR. The annotation of the 15 putative target transcripts showed an inverse expression pattern compared to their associated miRNAs at the same time points following chlorantraniliprole and flubendiamide exposure, except for a few positively correlated miRNA-mRNAs. For example, the relative expression of miRNAs (sfr-miR-novel-60, sfr-miR-10460-5p, sfr-miR-novel-11, sfr-miR-10476-5p) were significantly down-regulated, while the corresponding predicted targets (*CYB561D2*, *ChE1*, *ABCC13*, and *ABCA1*, respectively) were significantly upregulated following chlorantraniliprole treatments compared to the acetone-treated control at the same time points (Figs. 1 and 5). Furthermore, the expression of the target genes *JHBP* exhibited significant positive correlation for expression level with its corresponding miRNA sfr-miR-novel-10 at all-time points after exposure to chlorantraniliprole. Additionally, the putative targets of the miRNA, sfr-miR-novel-09 (*EcR-78C*), sfr-miR-novel-53 (*KLF 6_7* and *UGT2C1*), sfr-miR-10465-5p (*CYP6B2*, *GST*, *GST-2*, *UGT2B19*) and sfr-miR-10483-2-3p (*CYP12A2*, *RyR* and *BR-C*) showed variable relationships (either positive, negative or non-significant differences) with their potential targets after treatments of chlorantraniliprole at 12 and 24 h (Figs. 1 and 5).

**Figure 6:**
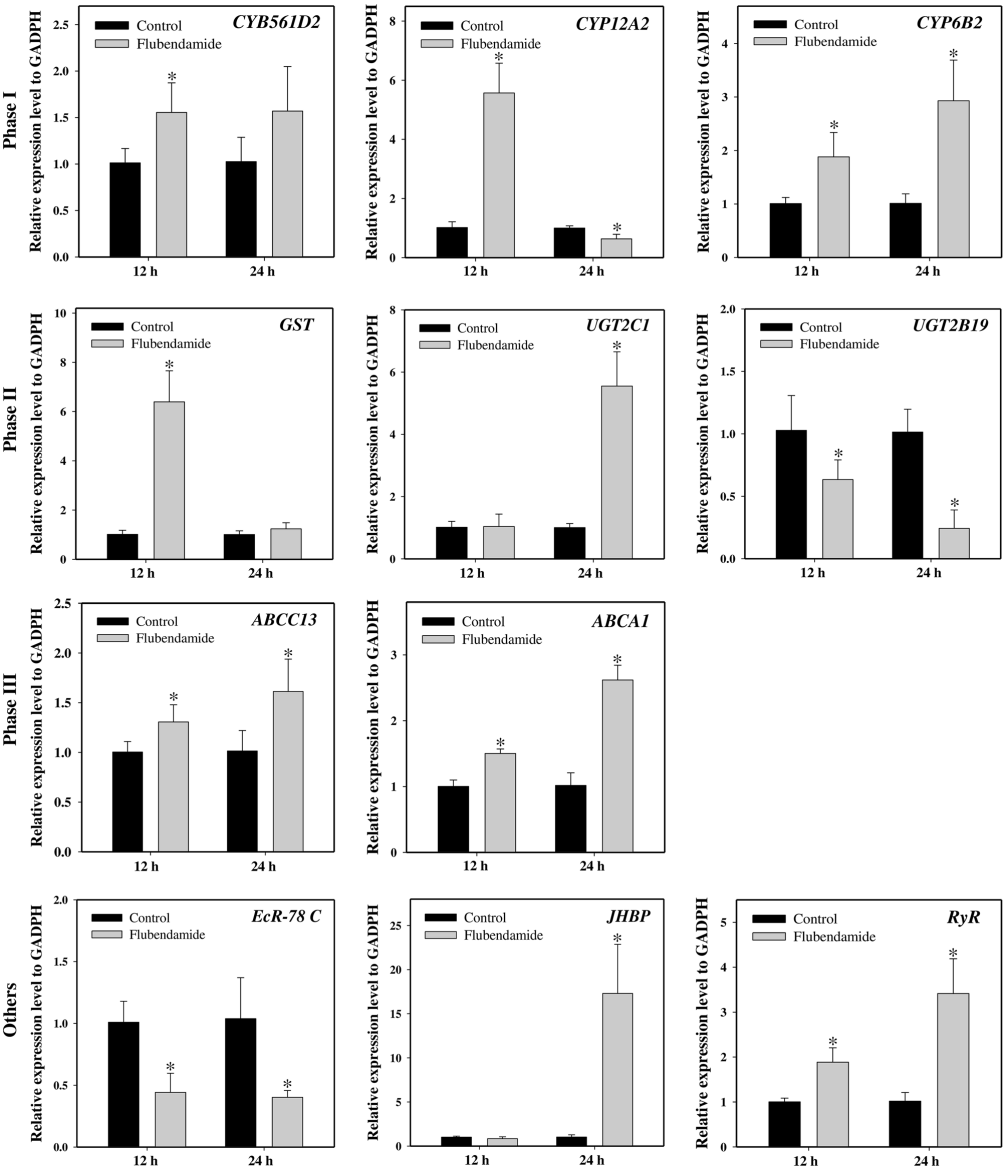
Relative expression of potential target genes of differentially expressed miRNAs after flubendamide treatment in the field populations of *S. frugiperda*. The expression levels were normalized by GADPH. Statistical significance was analysed using a paired t-test. The asterisks represent significant difference (P < 0.05).

Similarly, sfr-miR-10465-5p, sfr-miR-novel-11, and sfr-miR-10476-5p exhibited significantly decreased levels of expression with the significant upregulation of the corresponding predicted targets (*CYP6B2* and *GST*, *ABCC13* and *ABCA1*, respectively) in response to flubendamide exposure at all-time points, compared to the acetone-treated control (Figs. 2 and 6). Meanwhile, the expressions level of the potential targets for sfr-miR-novel-10 (*JHBP*) and sfr-miR-10465-5p (*GST-2* and *UGT2B19*) showed significant positive relationships with the expression of its corresponding miRNAs at 12 and 24 h in response to flubendamide exposure. Moreover, the expression levels of the putative target genes (*CYB561D2*, *ChE1*, *EcR-78C*, *KLF 6_7* and *UGT2C1*, *CYP12A2*, *RyR* and *BR-C*) and their corresponding miRNAs (sfr-miR-novel-60, sfr-miR-10460-5p, sfr-miR-novel-09, sfr-miR-novel-53, and miR-10483-2-3p, respectively) significantly demonstrated varying associations (i.e., positive, negative, or non-significant distinctions) subsequent to exposure to flubendamide at both the 12 and 24 h time points (Figs. 2 and 6). Overall, although the expression profiles of a few miRNAs and their target mRNAs showed no statistically significant correlations, mRNA expression was negatively associated with their corresponding miRNAs at the same time points after exposure to flubendiamide and chlorantraniliprole.

## Discussion

In recent years, there has been a growing interest among researchers in prediction of miRNAs which are involved in post-transcriptional regulation of insecticide resistance, particularly those targeting detoxification genes across various insect species^21^. However, understanding the profiles and functions of miRNAs in insecticide metabolism in *S*. *frugiperda* is significantly hindered, given the paucity of genetic functional validation and biochemical studies involving heterologously expressed enzymes for further investigation^28,47^. In this context, we have identified, characterized, and validated both conserved and novel miRNAs from *S*. *frugiperda* in response to diamide exposure. The discovery of these novel miRNAs will serve as a foundation for future research of miRNAs associated with diamide resistance in *S*. *frugiperda* and is a valuable contribution to the existing insect miRNA database. Subsequent analysis revealed potential target genes for some differentially expressed miRNAs that may play important roles in the development of insecticide resistance, providing insight into future approaches to integrated pest management.

Recent advances in high-throughput sequencing technologies and algorithms have enabled the precise identification of miRNAs and the prediction of their functions in several insect species, including *S*. *frugiperda*, with 404, 504, and 460 miRNAs^27–29^. The present study contributes to this existing knowledge by identifying 172 miRNA precursors, consisting of 109 known and 63 novel miRNAs, with the most abundant 22nt length miRNAs among all the libraries retained from resistant and susceptible strains of* S*. *frugiperda*. The typical length of the miRNAs obtained in our study was consistent with previous reports^21,28,31,38,48–50^. In addition, we found nine differentially expressed miRNAs in the resistant strain compared to the susceptible strain of* S*. *frugiperda*. Interestingly, our results demonstrated that seven miRNAs were significantly downregulated, and two were upregulated in the resistant strain compared to the susceptible strain of* S*. *frugiperda*.

Furthermore, this study identified the relative expression of nine differentially expressed miRNAs that exhibited significant downregulation (sfr-miR-novel-11, sfr-miR-10460-5p and sfr-miR-10476-5p) or upregulation (sfr-miR-novel-10) or displayed variable expression (sfr-miR-novel-09, sfr-miR-novel-60, sfr-miR-novel-53, sfr-miR-10465-5p, sfr-miR-10483-2-3p) at different time points following exposure to either chlorantraniliprole or flubendiamide in field populations of *S*. *frugiperda* collected from Korea. Overall, our expression analysis data showed a notable repression pattern of miRNAs and supported the hypothesis of miRNA-mediated post-transcriptional regulation in response to insecticide exposure. Consistent with our findings, some studies have provided evidence of repression of miRNAs (miR-7a, miR-8519, miR-2b-3p, and miR-14b-5p) conferring chlorantraniliprole resistance in *P*. *xylostella*^32,33^. Although we hypothesized that the differential miRNA expression patterns are associated with metabolic detoxification of diamides in *S*. *frugiperda*, the functional analysis, and verification of the regulatory relationships of these miRNAs remain to be elucidated.

Additional annotation of putative target transcripts of the nine differentially expressed miRNAs was predicted to target phase I (*P450* and *ChE1*), II (*GST* and *UGT*), III (*ABC*) detoxification pathways, and ryanodine receptor (*RyR*), which are likely to be involved in diamide detoxification. Several miRNA sequencing studies in insects have suggested that the induction of multiple cytochrome P450 genes (*P450*s), accompanied by the significant repression of their putative targeting miRNAs, may be a contributing factor to insecticide resistance^31,51,52^. Specifically, the reduced expression levels of several miRNAs (miR-8534-5p, miR-375-5p, miR-2b-3p, and miR-14b-5p) significantly increased the expression levels of putative P450 targets (*CYP6B6*, *CYP4G15*, *CYP9F2* and *CYP307A1*, respectively) that may play a role in metabolic resistance to chlorantraniliprole in *P*. *xylostella*^31,32^. Previous studies also support the hypothesis that the miR‑310s cluster induces the upregulation of several *CYP* genes associated with DDT resistance in *Drosophila*^36^. Furthermore, our results are consistent with a recent study speculating that miRNA-190-5p participates in resistance to chlorantraniliprole by upregulating *CYP6K2* in *S*. *frugiperda*^29^.

Comparative gene expression analysis in the current study revealed that the upregulation of cytochrome P450 target transcripts (*CYB561D2*, *CYP12A2*, and *CYP6B2*) was correlated with the downregulation of their corresponding miRNAs (sfr-miR-novel-60, sfr-miR-10483-2-3p, and sfr-miR-10465-5p, respectively), either after 12 or 24 h of treatment with chlorantraniliprole and flubendiamide in *S*. *frugiperda*. Previous studies indicated that the over expression of cytochrome P450s and reduced expression of miRNAs is associated with enhanced metabolic detoxification of insecticides and might contribute to insecticide resistance^29,32,53^. Despite the strong correlation between miRNAs and the significant upregulation of P450s in the present study, it is imperative to conduct further functional analyses to confirm the predicted results of miRNA-mediated post-transcriptional regulation in diamide detoxification.

Esterases (*EST*s) play vital roles as detoxification genes in insects. Here, we demonstrated that the target transcript *ChE1* was significantly upregulated with reduced expression of its corresponding miRNA (sfr-miR-10460-5p) in response to treatment with chlorantraniliprole. Previous studies have confirmed that the significant upregulation of esterase genes mediates resistance to malathion and chlorpyrifos in *B*. *dorsalis* and *P*. *xylostella*, respectively^54,55^.

Numerous studies have provided supporting evidence that the induction of multifunctional enzymes, such as GSTs and UGTs, by an insecticide usually increases the chances of involvement in the metabolism and detoxification of xenobiotic compounds^56^. The expression of *GSTe1* was upregulated when exposed to imidacloprid, highlighting its role in *Sogatella furcifera* resistance^53^. Likewise, overexpression of four GST genes, *NlGSTs1*, *NlGSTs2*, *NlGSTe1*, and *NlGSTm1*, was associated with imidacloprid resistance in *N*. *lugens*^56^. *tci-miR-1-3p* mediates cyflumetofen resistance by upregulating the expression of *TCGSTM4* in *T. cinnabarinus*^57^. The expression of *GSTu1* induced by miR-8525-5p is markedly involved in chlorantraniliprole resistance in *P*. *xylostella*^41^. Overexpression of *UGT2B17* significantly increased the toxicity of chlorantraniliprole in *P*. *xylostella*, suggesting that UGT plays a role in detoxification^42^. Hu et al.^58^ analysed that the three UGT genes (*UGT33J*3, *UGT33V4*, *UGT33T3*) were significantly induced. However, *UGT40U2* was slightly downregulated under chlorantraniliprole exposure in *S*. *exigua*. Similar results were found in the present study, whereby both positive and negative associations (upregulation or downregulation) of the expression levels of the miRNAs and their putative target genes, sfr-miR-novel-53 (*UGT2C1*) and sfr-miR-10465-5p (*GST*, *GST-2*, and *UGT2B19*), were observed in response to chlorantraniliprole and flubendiamide treatments.

To date, several studies have provided new insights into the involvement of ABC transporters in xenobiotic detoxification in arthropods^44,59–63^. Increased expression of multiple ABC transporters (*CsABCC8*, *CsABCG1C* and *CsABCH1*) was observed in the *C*. *suppressalis* after exposure to chlorantraniliprole^45^. The induction of numerous ABC transporters (*ABCG6*, *ABCG9* and *ABCG14*) confirms the regulation of chlorantraniliprole resistance in *P*. *xylostella*^46^. miR-998-3p has been reported to modulate the expression of *ABCC2* and mediate *Cry1Ac* resistance in *Helicoverpa armigera*, *S*. *exigua,* and *P*. *xylostella*^64^. The overexpression of *ABCB1* has been linked to the detoxification metabolism and chlorantraniliprole resistance in *S*. *frugiperda*^65^. Similar results were observed in our study, where two ABC transporters, *ABCC13* and *ABCA1*, were significantly upregulated with the downregulation of their corresponding miRNAs (sfr-miR-novel-11 and sfr-miR-10476-5p, respectively), indicating their participation in diamide detoxification. However, the underlying regulatory mechanisms require further investigation.

*RyRs* have been identified as a target of novel diamide insecticides in several insect species. However, the molecular mechanisms underlying *RyR*-mediated insecticide resistance remain unclear. Significantly downregulated expression of ryanodine receptors is associated with chlorantraniliprole resistance in *P*. *xylostella*^17^. Yang et al.^66^ provided empirical evidence regarding the roles of miRNAs in chlorantraniliprole insecticide resistance through the repression of ryanodine receptors in the white-backed planthopper *S. furcifera*. The upregulation of ryanodine receptor mRNA indicated increased resistance levels in *P*. *xylostella* and *S*. *exigua* in response to diamide exposure^67,68^. Interestingly, our results dovetail with the hypothesis that miRNAs, miR-7a and miR-8519, are most likely involved in chlorantraniliprole resistance through overexpression of *PxRyR* in *P*. *xylostella*^33^. The current study evaluated the expression level of the target transcript, ryanodine receptor (*RyR*), which is predicted to be the target of a known miRNA, sfr-miR-10483-2-3p. The expression profiles demonstrated the significant upregulation of the *RyR* gene with significant repression of the corresponding miRNA sfr-miR-10483-2-3p, establishing an inverse miRNA-mRNA relationship in* S*. *frugiperda* either at 12 and 24 h after chlorantraniliprole and flubendiamide exposure, respectively. However, it was positively correlated after 24 h of chlorantraniliprole treatment. Further investigations are required to confirm this hypothesis.

In insects, transcription factors (TFs), including Ecdysone receptor (*EcR*) and Broad-complex protein (*Br-C*), participate during metamorphosis and Krüppel-like factor 6_7 (*KLF6*_7) are associated with cellular growth and development. However, their exact role in insecticide resistance remains unclear. A previous study showed that *EcR* mRNA expression is significantly upregulated by methoxyfenozide in *S*. *frugiperda*^69^ and *S*. *exigua*^70^. Wu et al.^71^ reported the overexpression of *EcR* and *Br-C* genes associated with thiamethoxam resistance in the resistant strain of *A*. *gossypii*. The expression levels of *EcR* and *JHBP* genes were significantly upregulated in acetamiprid-resistant strains and downregulated in imidacloprid-resistant strains of *A*. *gossypii*^72,73^. The findings from this study showed that the increased expression of target genes involved in regulating the developmental process, such as *JHBP*, exhibited a positive relationship with the corresponding putative miRNA (sfr-miR-novel-10). In contrast, *Br*-*C* (sfr-miR-10483-2-3p) showed non-significant differences across all time intervals after both chlorantraniliprole and flubendiamide treatments. *EcR-78C* and *KLF6*_7 demonstrated variable relationships (negative or positive) with miRNAs (sfr-miR-novel-09 and sfr-miR-novel-53, respectively) at different time points after chlorantraniliprole and flubendiamide exposure.

Although there is currently no conclusive evidence to support specific roles of differentially expressed miRNAs in regulating corresponding target genes and their function in insecticide resistance, this study enticingly suggests the potential participation of miRNAs in coordinating detoxification responses during the diamide exposure or could point to their involvement in a regulation of transcript levels of their target detoxification genes in *S. frugiperda*.

## Methods

### *S. frugiperda* strains

The insecticide-resistant *S. frugiperda* strain was kindly provided by Dr. David Mota-Sanchez of Michigan State University (MSU) for developing miRNA libraries at MSU. A detailed description of this resistant strain has been provided in the literature^74,75^. The susceptible colony was obtained from the Benzon Research Lab (https://www.benzonresearch.com/). In the toxicity bioassays, the susceptible strain experienced 87% mortality when exposed to 3 ppm of chlorantraniliprole, whereas the resistant strain displayed a significantly lower mortality rate of 14% when exposed to 10 ppm of the same pesticide (Fig. S1). The *S. frugiperda* field population was collected from Yeongcheon, Republic of Korea (36°00′080′′ N, 128°98′862′′ E). All populations were maintained on an artificial diet at 25 ± 2 °C and 65 ± 5% relative humidity (RH) and a 14 h light (L): 10 h dark (D) photoperiod. Adults were provided with a 20% (w/v) sugar solution.

### Small RNA library construction and sequencing

Three biological replicates of 100 insecticide-resistant and -susceptible strains of 3–5 day-old larvae were collected for total RNA extraction using the Qiagen miRNeasy Mini Kit, according to the manufacturer’s instructions (Qiagen, Valencia, CA). The integrity and purity of the total RNA were assessed for all samples using an Agilent 2100 Bioanalyzer (Agilent Technologies, Germany), and RNA concentrations were estimated using NanoDrop One (Thermo, Wilmington, USA). Qualified total RNA was used to construct sRNA libraries using the TruSeq small RNA Sample Pre Kit (Illumina). Illumina sRNA libraries were constructed from each pool, and 50-bp single-end (SE50) sequence read data were generated on an Illumina HiSeq 4000 at the Research Technology Support Facility (Michigan State University, East Lancing, MI, USA). Clean reads were obtained from the raw data after processing adapters, low-quality reads, and sequences of fewer than 18 nucleotides using cutadapt v.3.4^76^. Clean reads were mapped to the *S*. *frugiperda* genome from the NCBI database (ZJU_Sfru_1.0) to analyse the distribution of the mapped sRNA on the reference sequence.

Furthermore, the trimmed reads were aligned to the miRNA precursors of *S*. *frugiperda*, *P. xylostella*, and *B. mori* in miRbase R.22. using miRDeep2^77^. Only tags matching exactly with the mature 5′ or 3′ regions of three previously annotated species miRNAs (miRbase R.21; file mature.fa) were accepted as known miRNAs and retained for further analysis. Unannotated clean sequences that did not match the above databases were further used to analyse and predict novel miRNAs using the miRDeep2 software.

### Differential expression analysis of miRNAs

To estimate the expression profiles within each replicate library from resistance (n = 3) and susceptible (n = 3) strains, the read counts of miRNAs were normalized to Reads Per Million mapped reads (RPM) through the normalization formula: Normalized expression = (number of mapped reads/total number of mapped reads) × 1,000,000. Then we used the package DESeq to analyse the differentially expressed miRNAs between libraries^78^, all *P* values were adjusted by Benjamini–Hochberg false discovery rate (FDR) procedure^79^, and the threshold for significant differential expression by default was P < 0.05 and |log2 (fold change)|> 1.

### Diamide toxicity bioassays

Bioassays using two diamide insecticides, chlorantraniliprole and flubendiamide, were conducted to calculate sublethal doses using field-collected third-instar larvae (6–7 days old) of *S. frugiperda* following the artificial diet-dipping method^80^. Briefly, test solutions of chlorantraniliprole (CAS No. 500008-45-7; ≥ 95% pure) and flubendiamide (CAS No. 272451-65-7; ≥ 98% pure) were prepared using technical grade insecticides (Sigma-Aldrich, St. Louis, MO, USA) dissolved in acetone to prepare the stock solution. Each treatment included four biological replicates, consisting of 10 individuals per replicate. The artificial diet was cut into an area of 1.5 cm^2^ with a thickness of 5 mm and dipped into the solutions for 30 s with acetone as the control. Artificial diets were air-dried at room temperature for 15 min. The larvae were starved for 12 h and then subjected to the treated artificial diet. The bioassays were kept at 25 ± 2 °C and 65 ± 5% RH under a photoperiodic regime of 14:10 (L:D). Mortality was assessed at 24 and 48 h after exposure to both diamide insecticides. Larvae were considered dead if they did not respond when nudged using a brush. LC_50_ values were calculated via probit analysis using SPSS software (version 26.0; IBM Corporation, United States).

### Inducible expression of miRNAs and corresponding target genes under diamide treatment

To systematically validate the sRNA sequencing estimated differences between the resistant and susceptible strains, we conducted differential expression analysis using RT-qPCR in the field population collected from the Republic of Korea. Furthermore, we analysed the relationship between the relative expression levels of the nine differentially expressed miRNAs and their potential target genes. Four biological replicates of 20 third-instar larvae were exposed to LC_50_ chlorantraniliprole and flubendiamide doses for 12 and 24 h. Live larvae were subjected to miRNA extraction using a miRNeasy Mini Kit. DNase I (Qiagen) was used to remove genomic DNA contaminants. First-strand cDNA was synthesized using the Mir-X miRNA First-Strand Synthesis Kit (Takara Bio USA, Inc.). RT-qPCR was performed with a Mir-X miRNA qRT-PCR TB Green kit (Takara Bio USA, Inc.) using miRNA-specific forward primers, according to the manufacturer’s instructions. All amplification reactions were run on a Light Cycler 96 Real-Time PCR system (Roche Diagnostics, Mannheim, Germany). The expression levels were calculated using the 2^-ΔΔCt^ method with U6 snRNA and GADPH as stable reference genes for miRNA and mRNA normalization, respectively^29,81,82^. Each qRT-PCR experiment was performed in three technical replicates and four biological replicates. The expression levels of each miRNA after diamide treatment were statistically compared using a paired t-test in SPSS software (version 26.0; IBM Corporation, United States). Statistical differences were considered significant at p < 0.05.

### Potential target gene prediction and functional annotation of differentially expressed miRNAs

Since miRNA functions by binding to their target genes, it is crucial to identify the putative targets of the differentially expressed miRNAs to annotate their role in diamide resistance. Potential target transcripts of miRNAs were predicted to be differentially expressed between resistant and susceptible strains using the psRNATarget server (https://www.zhaolab.org/psRNATarget/). The rules used for the target prediction were based on those suggested by Dai et al.^83^. In addition, the target gene ontology (GO) and corresponding pathways for all putative target transcripts were retrieved from Blast2Go (https://www.blast2go.com) and KAAS (https://www.genome.jp/kegg/kaas). We further visualized miRNAs and their corresponding target gene interactions using the Cytoscape Tool v3.9.1.

### Supplementary Information

Supplementary Figures.

Supplementary Tables.

## Data Availability

All data generated or analysed during this study are included in this article and its supplementary information files.
