# Supplementary material for: Identification of differentially expressed miRNAs associated with diamide detoxification pathways in Spodoptera frugiperda
Source: Sci Rep. 2024 Feb 21;14:4308. doi: 10.1038/s41598-024-54771-w (PMC10881993; doi:10.1038/s41598-024-54771-w)
Supplement: Supplementary file 1 — Supplementary Figures. [file 41598_2024_54771_MOESM1_ESM.pptx]

## Slide 1
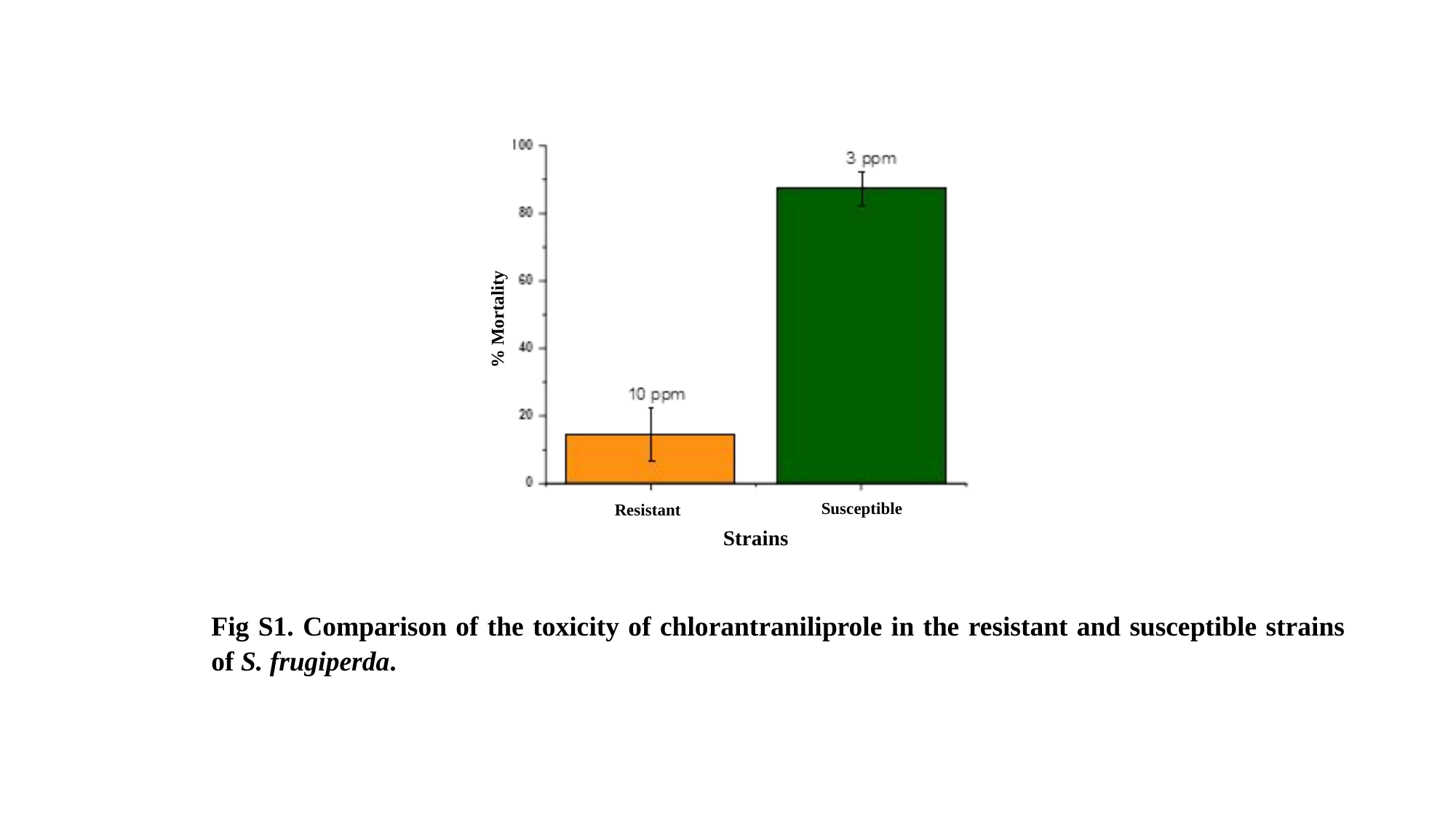

% Mortality
Susceptible
Resistant
Strains
Fig S1. Comparison of the toxicity of chlorantraniliprole in the resistant and susceptible strains of S. frugiperda.

## Slide 2
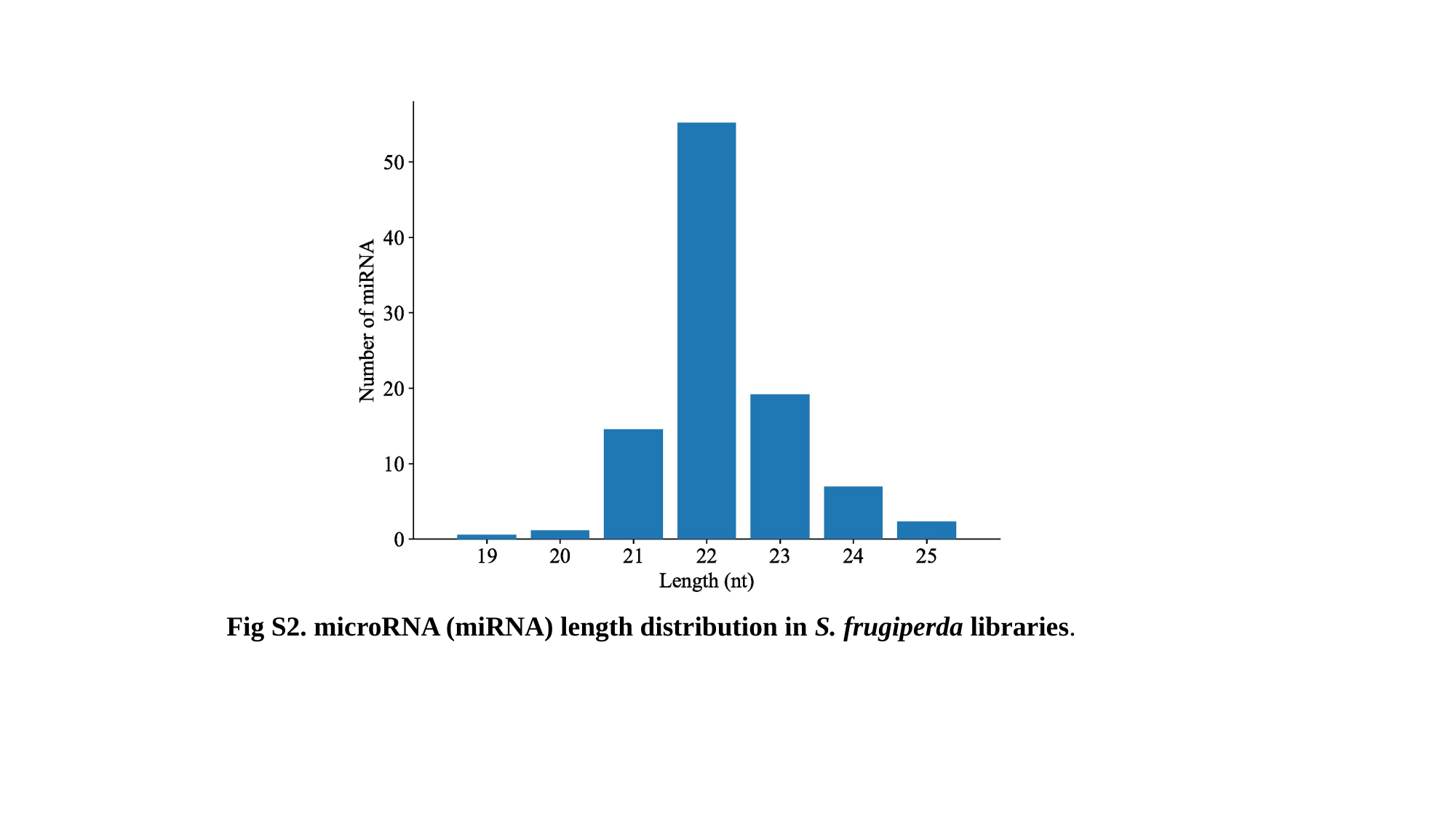

Fig S2. microRNA (miRNA) length distribution in S. frugiperda libraries.

## Slide 3
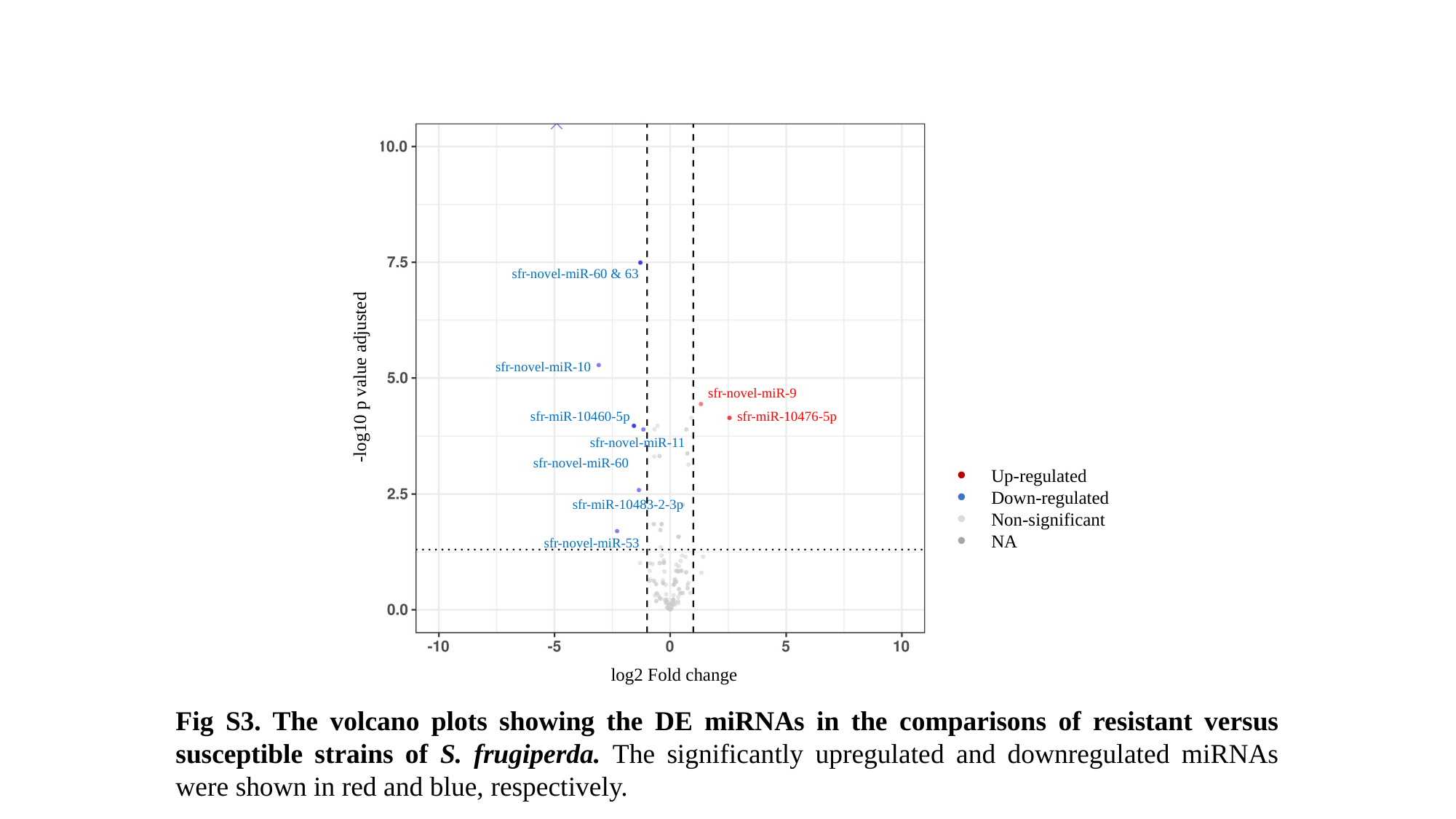

-log10 p value adjusted
Up-regulated
Down-regulated
Non-significant
NA
log2 Fold change
sfr-novel-miR-60 & 63
sfr-novel-miR-10
sfr-novel-miR-9
sfr-miR-10476-5p
sfr-miR-10460-5p
sfr-novel-miR-11
sfr-novel-miR-60
sfr-miR-10483-2-3p
sfr-novel-miR-53
Fig S3. The volcano plots showing the DE miRNAs in the comparisons of resistant versus susceptible strains of S. frugiperda. The significantly upregulated and downregulated miRNAs were shown in red and blue, respectively.

## Slide 4
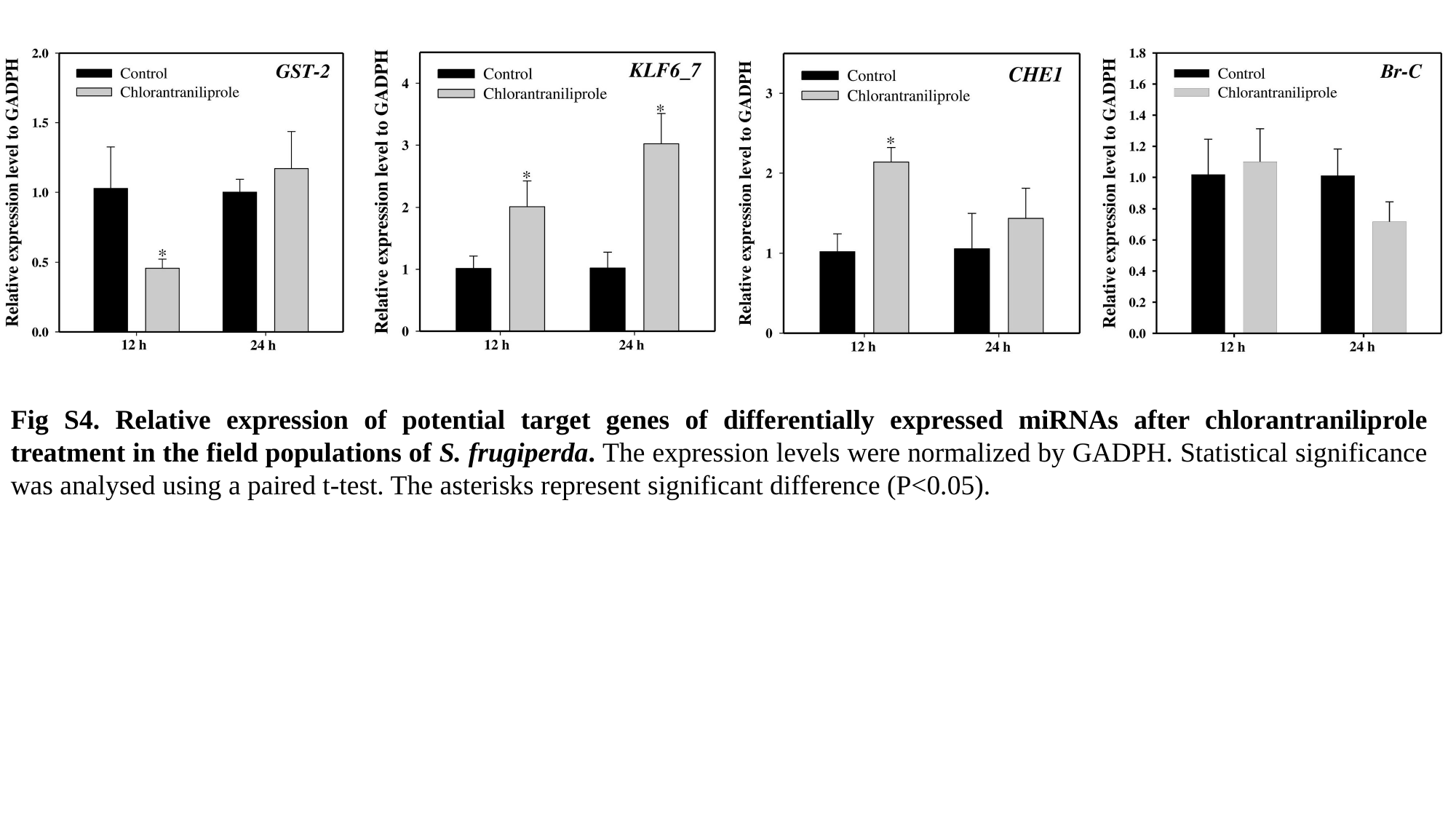

Fig S4. Relative expression of potential target genes of differentially expressed miRNAs after chlorantraniliprole treatment in the field populations of S. frugiperda. The expression levels were normalized by GADPH. Statistical significance was analysed using a paired t-test. The asterisks represent significant difference (P<0.05).

## Slide 5
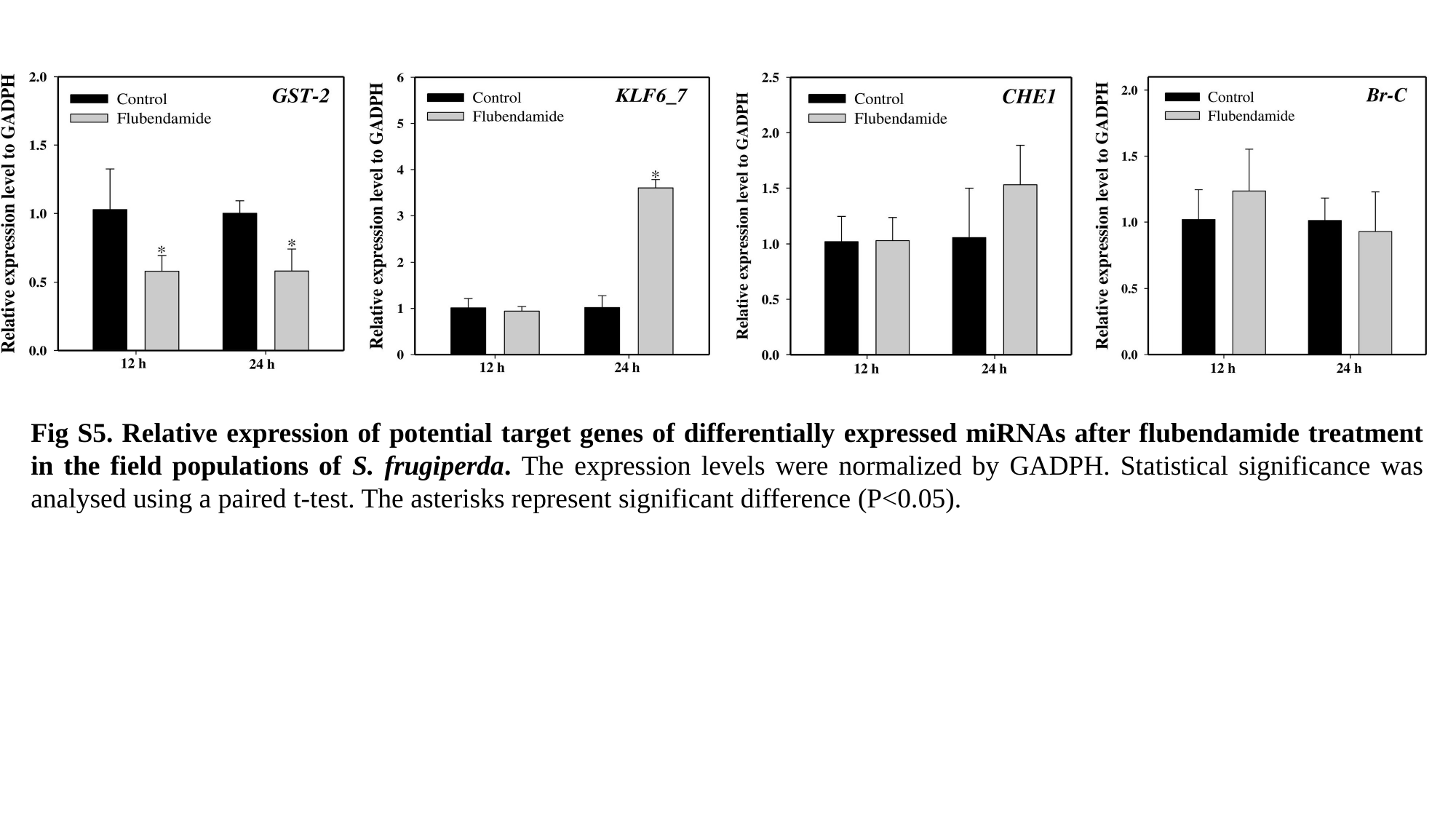

Fig S5. Relative expression of potential target genes of differentially expressed miRNAs after flubendamide treatment in the field populations of S. frugiperda. The expression levels were normalized by GADPH. Statistical significance was analysed using a paired t-test. The asterisks represent significant difference (P<0.05).
